# The application of evidence-based measures to reduce surgical site infections during orthopedic surgery - report of a single-center experience in Sweden

**DOI:** 10.1186/1754-9493-6-11

**Published:** 2012-06-14

**Authors:** Annette Erichsen Andersson, Ingrid Bergh, Jón Karlsson, Bengt I Eriksson, Kerstin Nilsson

**Affiliations:** 1 University of Gothenburg, The Sahlgrenska Academy, Institute of Health and Care Sciences, Gothenburg, Sweden; 2 Department of Anesthesia, Surgery and Intensive Care, Sahlgrenska University Hospital, Gothenburg, Sweden; 3 University of Skövde, School of Life Sciences, Skövde, Sweden; 4 Department of Orthopedics, Sahlgrenska University Hospital, Gothenburg, Sweden; 5 University of Gothenburg, The Sahlgrenska Academy, Institute of Clinical Sciences, Gothenburg, Sweden; 6 Department of Anesthesiology/Surgery, Sahlgrenska University Hospital/Östra, Smörslottsgatan 1, Gothenburg, SE-416 85, Sweden

## Abstract

**Background:**

Current knowledge suggests that, by applying evidence-based measures relating to the correct use of prophylactic antibiotics, perioperative normothermia, urinary tract catheterization and hand hygiene, important contributions can be made to reducing the risk of postoperative infections and device-related infections. The aim of this study was to explore and describe the application of intraoperative evidence-based measures, designed to reduce the risk of infection. In addition, we aimed to investigate whether the type of surgery, i.e. total joint arthroplasty compared with tibia and femur/hip fracture surgery, affected the use of protective measures.

**Method:**

Data on the clinical application of evidence-based measures were collected structurally on site during 69 consecutively included operations involving fracture surgery (n = 35) and total joint arthroplasties (n = 34) using a pre-tested observation form. For observations in relation to hand disinfection, a modified version of the World Health Organization hand hygiene observation method was used.

**Results:**

In all, only 29 patients (49%) of 59 received prophylaxis within the recommended time span. The differences in the timing of prophylactic antibiotics between total joint arthroplasty and fracture surgery were significant, i.e. a more accurate timing was implemented in patients undergoing total joint arthroplasty (*p* = 0.02). Eighteen (53%) of the patients undergoing total joint arthroplasty were actively treated with a forced-air warming system. The corresponding number for fracture surgery was 12 (34%) (*p* = 0.04).

Observations of 254 opportunities for hand hygiene revealed an overall adherence rate of 10.3% to hand disinfection guidelines.

**Conclusions:**

The results showed that the utilization of evidence-based measures to reduce infections in clinical practice is not sufficient and there are unjustifiable differences in care depending on the type of surgery. The poor adherence to hand hygiene precautions in the operating room is a serious problem for patient safety and further studies should focus on resolving this problem. The *WHO Safe Surgery checklist* “time out” worked as an important reminder, but is not per se a guarantee of safety; it is the way we act in response to mistakes or lapses that finally matters.

## Background

Given that deep surgical site infections (SSI) following orthopedic implant surgery result in the drainage of community and hospital resources [1-3], every possible measure should be taken to reduce potential risk factors associated with SSI. In addition, these infections also cause major suffering in patients [4]. The development of an SSI is a complex process dependent on several different interacting properties and prerequisites related to the patient, the surgical environment, including staff behavior, and finally the surgical technique. For this reason, the measures taken to reduce the risk of infection need to be directed towards all these areas. Current knowledge suggests that, by applying evidence-based measures during surgery, major contributions can be made in reducing the risk of SSI and device–related infections (DRI). This includes securing the correct timing of prophylactic antibiotics [5], maintaining intraoperative normothermia during surgery [6,7], avoiding the inadequate use of urinary tract catheterization (UTC) [8,9] and, above all, adhering to basic hand hygiene precautions [10]. In order to succeed, all the members of the operating room (OR) team, including anesthetic nurses and physicians, need to have scientific knowledge on how this can be accomplished. In this study, we therefore focus on the potential for risk reduction within anesthetic care.

The aim of this study was to explore and describe the application of intraoperative evidence-based measures designed to reduce the risk of SSI and DRI during orthopedic implant surgery. In addition, we aimed at investigating whether the type of surgery, i.e. total joint arthroplasty (TJA) compared with fracture surgery (internal fixation with osteosynthesis or a hemi-prosthesis) (FS), affected the use of protective measures.

## Methods

### Setting

The study was set in a Swedish orthopedic teaching hospital performing approximately 10,000 surgical procedures a year. In 2009, the hospital participated in a national quality improvement project (PRISS – prosthetic joint infections must be stopped) [11], based on a collaborative effort between several professional societies aiming to reduce the incidence of SSI in relation to prosthetic joint surgery. The routines for and implementation of SSI prophylactic measures at every participating hospital were reviewed and evaluated by peers. The result was handed over to the hospital management team, which set up a multidisciplinary task force to address the areas identified as being in need of improvement. They included the air quality in the OR and the appropriate timing, dose and type of prophylactic antibiotic drug. In the same year, the *WHO Safe Surgery checklist*[12] was also implemented.

### Observational methods

Data were collected at a total of 69 consecutively included operations involving FS (n = 35) and TJA (n = 34) during the daytime and, in most of the cases, once a week, over a twelve-month period from April 2010 to May 2011; i.e. one year after the PRISS project was initiated and the WHO checklist was implemented.

All the data were collected onsite by a trained, experienced observer (AEA) using a pre-tested structured observation form. The number of observations varied in relation to the different studied variables. This variation was due to the fact that all the variables were not available for observation during all 69 surgical procedures. See Table 1 for all the included variables and the number of observations. The variables were included on the basis of scientific evidence for risk reduction in relation to infections. Moreover, the selected measures should also be well known to the OR staff and possible for the non-scrubbed members of the OR team to apply. The OR teams were aware that a study of infection control was being carried out, but they were not aware of exactly which items were of interest in this study. Observations took place in 6 parallel ORs and the adjacent preparation rooms. Three of the ORs were equipped with vertical parallel airflow ventilation systems (LAF) and 3 with displacement ventilation systems.

**Table 1:** Included variables

**Included variables**	**Numbers of observations**
***Basic data***	
Type of surgery	69
Length of surgery	69
ASA classification score^1^	68
Use of WHO checklist (“time out”)	69
***Preventive measures in relation to:***	
*Prophylactic antibiotics*	
Type	68
The difference in minutes between completed infusion and incision (or application of tourniquet)	59
*Normothermia*	
Method used for monitoring body temperature	69
Method used for maintaining normothermia	68
*UTC*	
Adherence to aseptic insertion technique	11
The use of an indwelling urinary tract catheter	66
*Air cleanliness*	
All hair covered by a surgical hood?	66
*Transmission of micro-organisms*	
Adherence to hand hygiene guidelines	
Correct use of protective gloves	254

^1^ASA Physical Status Classification System [53].

ASA Physical Status 1 - A normal healthy patient.

ASA Physical Status 2 - A patient with mild systemic disease.

ASA Physical Status 3 - A patient with severe systemic disease.

ASA Physical Status 4 - A patient with severe systemic disease that is a constant threat to life.

UTC: Urinary tract catheterization.

The implementation of the WHO Safe Surgery checklist has been associated with improvements in surgical outcome and reduced postoperative complications [12]. The original checklist consists of 19 items to be orally confirmed by the OR team. It is used at three critical transitional phases in care, before anesthesia, just prior to incision and before the patient is taken out of the OR. In this study, we focused on observations in relation to the second phase called “time out” and, more specifically, the administration of prophylactic antibiotics. The local guideline states that the patients should receive a completed infusion of prophylactic antibiotics 30 min prior to surgery. Cloxacillin is recommended as the first-line treatment/prophylaxis and three doses should be given within 24 h of surgery. The first dose is to be given as an infusion by the anesthetic nurse. Data on the timing of antibiotic prophylaxis were to be retrieved from patient records. However, during the initial onsite observations, it was noted that a discrepancy of approximately 5 to 25 min existed between the actual times of completed infusion and the times registered in the patient records. In addition, the time of administration was found to be an inaccurate measurement of timing as the infusions could last from approximately 15 min to about one hour. It was subsequently decided that these data had to be recorded after direct observations of completed infusion to ensure accuracy. Achieving optimal tissue levels at the time of incision has been shown to be crucial [13]. Current knowledge suggests that this is approximately 30 min before incision in relation to the type of antibiotics with a half-life of 30 min [14,15]. Based on this, infusions given 45–15 min prior to surgery or the application of a tourniquet were considered to be within an acceptable time span.

According to local guidelines, perioperative UTC should only be used for strict indications, such as an estimated length of surgery of > 2.5 h or renal insufficiency.

Hand hygiene in the OR was monitored using a modified version of the World Health Organization hand hygiene observation method [16] and in accordance with the Swedish national guidelines stating that hand disinfection (with an alcohol-based hand rub) must be carried out before and after every treatment, care or direct contact with a patient and before and after the use of protective gloves [17]. Using a single observer meant that it was necessary to select the items that were going to be observed, as one observer cannot perform comprehensive observations including all the events in the OR. We chose to observe hand disinfection and glove use in relation to invasive procedures such as peripheral venous catheter, arterial line, urinary catheter, regional anesthesia and tracheal intubation. Observations of hand disinfection prior to opening and handing over sterile material (such as implants) to the scrub nurse were also included. Observations of the risk of hand transmission of microorganisms were recorded. For example, if, after tracheal intubation, no hand disinfection was applied and the observed individual subsequently touched a clean site such as stopcocks, this was recorded as a risk for transmission of microorganisms. In addition to structured observations, field notes were taken throughout the study period in order to capture talks and events in relation to the study variables.

### Data analysis

Data were analyzed by descriptive statistics. For comparisons of continuous variables between groups, independent sample t-tests were used, reporting mean, SD and 95% confidence intervals (CI). For examinations of categorical data, we used chi-square tests of independence with Yates’ Correction for Continuity (for 2 by 2 tables). Significance was defined as *p* < 0.05 and all the tests were two-tailed. Comparisons between groups were not initially a part of the study protocol and the statistical power was therefore calculated on the basis of the mean values and SD for the timing of prophylactic antibiotics measured in minutes and actual sample size. Using an alpha error level of 5% gave a statistical power of 75%.

In relation to hand hygiene, opportunities for hand disinfection represented the level of analysis. Adherence was calculated by dividing the number of applications of hand disinfection by the total number of opportunities. A hand hygiene opportunity was defined as a situation requiring hand disinfection. A hand hygiene application was defined as the use of an alcohol-based hand rub in relation to an opportunity. The amount of product used and the duration of application were not recorded. Adherence was stratified by professional category and indication. Sample size calculations for the number of hand hygiene opportunities were performed in order to have an opportunity to compare an adherence in two time periods. With an anticipated 20% adherence at baseline, the possibility of detecting a 15% difference before and after an intervention would require a sample size of 250 observed opportunities per time period. Manifest content analysis was applied to data derived from field notes [18].

### Ethics

The study was approved by the Ethics Committee in Gothenburg, Sweden (Dnr: 157–10). Written and oral information was given in line with the four principal requirements of the Helsinki Declaration, autonomy, beneficence, non-malfeasance and justice [19]. Accordingly, informed consent was obtained from all the OR teams prior to observations.

## Results

We observed a tendency towards higher ASA scores among patients undergoing FS compared with TJA, (Table 2). There were no significant differences in the length of surgery measured in minutes between the groups (FS: m = 85.6, SD 41) and (TJA: m = 99.3 SD 28), *p* = 0.12.

**Table 2:** Distribution of ASA score within type of surgery

**Type of surgery**	**ASA score**				**Total**
	**1**	**2**	**3**	**4**	
Fracture surgery	9	15	8	2	34
TJA	12	18	4	0	34
Total	21	33	12	2	68

### Prophylactic antibiotics

The administration of prophylactic antibiotics was observed during 30 FS and 30 TJA operations. One fracture operation was removed from the analysis, as this patient had received antibiotic treatment for more than 24 h before the operation and it could therefore not be considered to be prophylactic. In all, 29 patients (49%) of 59 received prophylaxis within the recommended time (45–15 min before incision or the application of a tourniquet).

With regard to FS, 12 patients received their prophylaxis within the recommended time span. Two patients received prophylaxis >45 min before incision, whereas 5 received their prophylaxis 2–14 min before incision. Ten patients received their prophylaxis after incision or the application of a tourniquet.

In the TJA group, no patients received prophylaxis after incision. Seventeen received prophylaxis within the recommended time span. Ten patients received prophylaxis 0–14 min prior to incision and 3 >45 min prior to incision.

**Figure 1:**
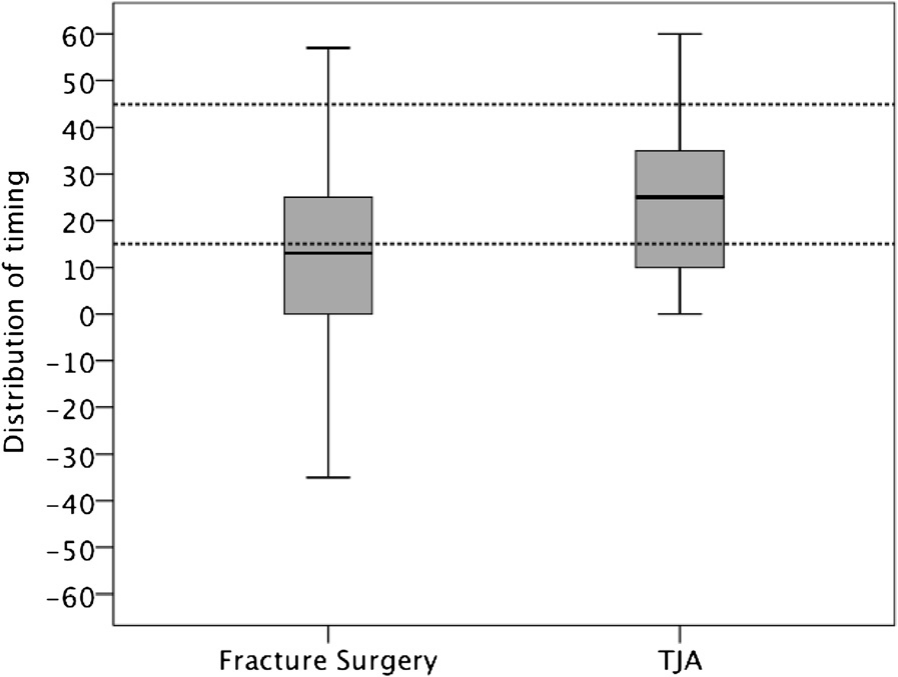
Timing of Prophylactic Antibiotics in relation to type of surgery within recommended timespan (15-45 min) prior to incision.

The mean time for prophylaxis in the TJA group was 24 min before incision (SD 15.9, 95% CI 18.0-29.9, range; 0–60 min), while the mean time for FS was 13.2 min (SD 21.6, 95% CI 4.9-21.4, range: -35-57 min). This difference between TJA and FS was significant (*p* = 0.03), (Figure 1). For results relating to the type of antibiotics used, see Table 3.

**Table 3:** Type of prophylaxis in relation to type of surger0079

	**Fracture surgery**	**TJA**
**Cloxacillin**	28 (80%)	32 (94.1%)
**Clindamycin**	3 (8.6%)	2 (5.9%)
**Cefuroxim**	2 (5.7%)	0
**Cefotaxim**	1 (2.9%)	0

### “time out”

The WHO Safe Surgery checklist “time out” was applied during a total of 45 surgical procedures (in 28 out of 35 FS procedures and 17 out of 34 TJA procedures).

Field notes revealed that the use of the WHO Safe Surgery checklist was a well-integrated, accepted practice, causing no notable objections among the surgical team members when used. In cases where prophylactic antibiotics had not been administered at the “time out”, the checklist worked as a reminder. The reasons for prophylaxis not being administered were; antibiotics had not been prescribed, the anesthetic nurse forgot to administer the drug or the prescription was not available due to administrative problems with the computerized medical notes. When prophylaxis had not been completed prior to incision or the application of a tourniquet, this was only rarely communicated to the surgeon. In those cases in which the surgeons received information on inadequate timing, it generally resulted in no further action and the surgical procedure was initiated with an incision or the application of a tourniquet.

### Urinary tract catheterization

When it came to the intraoperative use of UTC, 20 (57%) of the patients undergoing FS and 15 (43%) of those who underwent TJA received a catheter during the preoperative period. This difference was not statistically significant, *p* = 0.46.

The technique for catheterization was observed in 11 cases. In 10 of 11 cases, no hand disinfection was carried out by the person who inserted the catheter and, in 6 of 11 cases; no hand disinfection took place after completed insertion. In Table 4, the distribution of UTC use in relation to ASA classification score is shown.

**Table 4:** Use of UTC in relation to ASA score and type of surgery

**ASA score**	**UTC in FS**^**1**^	**UTC in TJA**^**2**^
**1**	2 (22.2%)	4 (36.4%)
**2**	8 (57.1%)	8 (47.1%)
**3**	7 (87.5%)	3 (75%)
**4**	2 (100%)	0

^1^ Urinary tract catheterization in fracture surgery.

^2^ Urinary tract catheterization in total joint arthroplasty.

### Intraoperative normothermia

Only 3 (8.6%) of the patients undergoing FS were monitored for body temperature. The majority of patients were (91.4%) operated on in rooms equipped with a conventional/displacement ventilation system with a mean room temperature of 21°C. Twelve (34.3%) of these patients were actively treated with a forced-air warming system. Nineteen (54.3%) were given a thin cotton quilt, whereas, in 4 cases (11.4%), active warming systems were applied approximately one hour after incision.

The corresponding numbers for the TJA group were 5 (14.7%) for monitoring body temperature, 18 (52.9%) for active treatment, 9 (26.5%) for passive/cotton quilt and 6 (17.6%) for the later application of an active warming system. All patients undergoing TJA were operated on in an operating room equipped with a parallel airflow ventilation system maintaining a mean room temperature of 19°C.

The application of any (both initial and later) forced-air warming system differed significantly between the groups (*p* = 0.04).

### Air quality

During 66 surgical procedures, we observed adherence to the practice of keeping all hair covered by a surgical hood. In 14 (20%) of the cases, one or more individuals in the OR team had their hair hanging outside the hood during surgery. There was no significant difference between groups.

### Hand disinfection

A total of 254 opportunities for hand hygiene were observed during 10 observational sessions. Most opportunities for observations of invasive procedures typically occur before and during the induction phase and before the surgical incision. For results, see Table 5.

**Table 5:** Adherence in percent to hand disinfection guidelines before and after hygiene opportunities

	**Before**	**After**	**Total (n**^**1**^**)**
***Invasive procedure***	6.2%	17.7%	226
***Handling sterile products***	7.1%		28
***Adherence/professional category***			
Anesthesiologist	6.5%	3.7%	58
Anesthetic nurses	1.5%	10.3%	136
Nurse assistants	13.9%	27.8%	72
Surgical nurses	2^3^	4^3^	6^3^
***Use of non-sterile protective gloves***	**Yes (clean)**	**Yes (used**^2^**)**	
	30.3%	19.2%	132
***Risk of transmission of micro-organisms***		76.6%	141

^1^ Total number of observations.

^2^ Gloves already being used prior to the invasive procedure.

^3^ Very low numbers of observations.

## Discussion

The most important findings in the present study were that evidence-based measures for preventing SSI during anesthetic care were not sufficiently implemented Furthermore, differences in the quality of care appear to exist between patients undergoing TJA and patients undergoing FS. These differences cannot be justified, especially since we know that fracture patients are more susceptible to infection. Orthopedic trauma patients suffer from preoperative soft-tissue and skeletal damage, along with co-morbidities and minimal opportunities for preoperative optimization, which have been shown to be major risk factors for this group of patients [20]. A trend towards higher ASA classification scores, which are per se associated with a higher risk of SSI, was also demonstrated in our study [21]. An overall risk assessment of the trauma patient should lead to meticulously applied risk reduction measures during anesthetic care. Among hip and knee arthroplasty surgeons, there is a strong tradition of research on how SSI can be prevented [22-24] and the quality of care is thoroughly monitored [25]. The national PRISS project could also be seen as a reflection of this interest. However, in the area of orthopedic trauma surgery, there are more limited data on preventive measures and risk factors [20], along with reports on relatively high infection rates, 4.2% [2], 5.2% [26], 6.9% [27]. This high SSI rate could at least partly be explained by differences in the quality of care in relation to infection control observed in this study between TJA and FS.

We found that more favorable conditions were created for TJA patients during surgery. They were all operated on in operating rooms equipped with laminar airflow systems, designed to reduce the number of colony forming units (CFU) to well below 5/m^3^. Fracture patients, on the other hand, had their procedures performed in displacement-ventilated ORs (91.4%). A recent study carried out in the same displacement –ventilated ORs and based on 116 active air samples demonstrated that the mean CFU/m^3^ values exceeded the recommended levels for orthopedic surgery, < 10 CFU/m^3^ (m = 15.9, SD 13.4 CI 13.1-18.7) [28]. One of the basic prerequisites for safe surgery in orthopedics is optimal air quality [23,29,30]. The dispersal of particles from the individuals present in the OR is considered to be the most important source of airborne contamination and, for this reason, the non-scrubbed staff can reduce airborne contamination by observing the correct clothing regimen and by wearing surgical hoods that cover all their hair [31-33]. In 14 of 66 procedures, it was observed that OR staff had hair hanging outside the surgical hood, a fact that can adversely affect air quality and, as a result, patient safety.

Systematic reviews strongly support the importance of the optimal timing of antibiotic prophylaxis in relation to TJA, as well as fracture surgery, stating that, for every 13 patients who are treated, one wound infection would be prevented [34,35]. In the present study, only 47% of the patients received prophylaxis within the recommended time span. Similar results (45-57%) have been reported by Stefansdottir et al. [36]. In eight cases, other types of prophylaxis then Cloxacillin were administrated. This raises the question on if it is manageable in clinical practice to have different guidelines depending on type of prophylaxis and their half-life. One interesting observation in the present study was that none of the patients in the TJA group had a major violation of the recommended timing, i.e. received prophylaxis after incision or the application of a tourniquet, whereas 10 of 29 patients undergoing fracture surgery had their antibiotics after the start of surgery. The timely administration of prophylactic antibiotics is of the utmost importance, as a study of 1992 patients undergoing total hip arthroplasty showed that those who received prophylaxis after incision had the highest odds of developing an SSI [37]. The WHO checklist did, in fact, function as an important reminder, but, as we discovered, the checklist per se is not a guarantee of safety; it is instead the way we react to mistakes or lapses that finally matters.

Clear evidence has been presented of the relationship between SSI and mild hypothermia and accordingly the protective effect of normothermia during surgery [6,7]. The clinical setting in our hospital, with fairly cold ambient air (19-21°C) in combination with the patient’s impaired thermoregulatory system caused by regional or general anesthesia [38], supports the use of an active patient warming system. Even mild perioperative hypothermia has been shown to produce a series of adverse effects in patients undergoing surgery. It is associated with an increased risk of blood loss and blood transfusion [39], as well as a risk of increased cardiac morbidity [40], altered drug metabolism [41] and prolonged hospitalization [7]. Questions have been raised whether these warming systems could actually be vectors of infection, but studies have shown that this is not the case [42,43].

Urinary tract infection (UTI) is the most common healthcare-associated infection and a frequently observed complication after major joint surgery [44]. In hospital settings, almost all these infections develop as a result of urinary tract catheterizations [9]. It has been demonstrated that catheter-related UTI contributes to an increased length of stay, costs, morbidity and excessive antimicrobial drug use [45]. However, the management of the UTC and length of time it is used, influences the development of a UTI. We found that the use of UTC increased with increasing ASA-classification score, which is not surprising as this reflects the patients’ health status. In patients with an ASA score of 3 or 4, the use of UTC is not only justified but also most frequently necessary. Even so, on the basis of our results, we draw the conclusion that more could be done to avoid its use in healthy patients, when the estimated length of surgery does not exceed 2.5 h. However, the most worrying finding was the poor compliance with the practice of using an aseptic insertion technique. In 10 of 11 directly observed insertions of UTC, the OR staff did not perform hand disinfection before the insertion and, in 6 of 11 cases, they did not even do so after the insertion. These results are linked to poor adherence (11.9%) to hand disinfection guidelines, resulting in bacterial transmissions observed in the OR. Recent studies in the UK [46] and the USA [47,48] presenting similar results indicates that this is an international problem that needs to be resolved. The reasons behind low adherence to the different clinical guidelines in this study is in line with consisting findings of the gap between evidence and practice in health care [49]. Producing standard protocols and guidelines will not per se result in enhanced patient safety [50] Hence; the complexity of implementing guidelines and behavior change should not be underestimated as adoption of a guideline depends on many different factors. Obstacles for successful implementation could be found on individual, structural and cultural levels. In addition, we also need to take in account the many different and competing demands health care professionals meet in every day practice [51]. By extracting knowledge from the implementation science, it is possible that we could gain deeper insight in how to select the appropriate strategies for implementation of guidelines in the surgical environment.

### Limitations

Observational studies could be susceptible to bias [52]. Human perceptual errors could affect the information that is obtained, together with behavioral distortion due to the presence of an observer. Several measures were taken to address potential bias. Firstly, the observational form was pre-tested and modified, secondly, the observer had no prior connection with the ward under observation and, thirdly, the observer underwent self-training sessions to maximize accuracy. The staff was also blinded to exactly what was being observed. Concealed observations to reduce reactivity were not feasible and were also considered to be a possible source of distrust between the OR staff and the observer.

One limitation of this study was that comparisons between groups were not included in the initial study protocol, resulting in an estimated statistical power of 75%.

## Conclusions

There are unjustifiable differences in care and surgical conditions between patients undergoing TJA and fracture surgery. We conclude that the same standards and routines that have become a natural part of the safety culture in relation to TJA would be beneficial to patients undergoing fracture surgery and most probably result in improved surgical and patient outcomes. It is time for a change of perspectives, leading to safer care for trauma patients, which requires a more overarching discussion of our priorities in this field. In order to implement a paradigm shift, intervention studies are needed to support a change of this kind. Moreover, the results of the current study indicate that the utilization of evidence-based measures to reduce SSI and HAI in clinical practice is not enough; much more could be done to prevent SSI during both TJA and fracture surgery. So, by taking benefit of the opportunities during anesthetic care, important contributions can be made in creating a safer surgical environment, which would be an active counterweight to inherent risk factors. The poor adherence to hand hygiene precautions in the OR is a serious problem for patient safety and further studies should focus on resolving this problem.

## Competing interests

The authors declare that they have no competing interests.

## Authors’ contributions

AEA, IB, BE, JK, and KN designed the study. AEA collected all data. IB and AEA performed statistical analyses. The manuscript was prepared by AEA, IB, BE, JK, and KN. All authors read and approved the final version of the manuscript.

## References

[B1] Bozic KJ Ries MD The impact of infection after total hip arthroplasty on hospital and surgeon resource utilization J Bone Joint Surg Am 2005 87 8 1746 1751 10.2106/JBJS.D.02937 16085614

[B2] Coello R Charlett A Wilson J Ward V Pearson A Borriello P Adverse impact of surgical site infections in English hospitals J Hosp Infect 2005 60 2 93 103 10.1016/j.jhin.2004.10.019 15866006

[B3] de Lissovoy G Fraeman K Hutchins V Murphy D Song D Vaughn BB Surgical site infection: incidence and impact on hospital utilization and treatment costs Am J Infect Control 2009 37 5 387 397 10.1016/j.ajic.2008.12.010 19398246

[B4] Andersson AE Bergh I Karlsson J Nilsson K Patients' experiences of acquiring a deep surgical site infection: An interview study Am J Infect Control 2010 38 9 711 717 10.1016/j.ajic.2010.03.017 21034980

[B5] Polk HC Jr Christmas AB Prophylactic antibiotics in surgery and surgical wound infections Am Surg 2000 66 2 105 111 10695738

[B6] Melling AC Ali B Scott EM Leaper DJ Effects of preoperative warming on the incidence of wound infection after clean surgery: A randomised controlled trial Lancet 2001 358 9285 876 880 10.1016/S0140-6736(01)06071-8 11567703

[B7] Kurz A Sessler DI Lenhardt R Perioperative normothermia to reduce the incidence of surgical-wound infection and shorten hospitalization. Study of Wound Infection and Temperature Group N Engl J Med 1996 334 19 1209 1215 10.1056/NEJM199605093341901 8606715

[B8] Stephan F Sax H Wachsmuth M Hoffmeyer P Clergue F Pittet D Reduction of urinary tract infection and antibiotic use after surgery: a controlled, prospective, before-after intervention study Clin Infect Dis 2006 42 11 1544 1551 10.1086/503837 16652311

[B9] Gould CV UC Agarwal RK Kuntz G Pegues DA Guideline for prevention of catheter-associated urinary tract infections 2009 Infect Control Hosp Epidemiol 2010 31 4 319 326 10.1086/651091 20156062

[B10] Pittet D Compliance with hand disinfection and its impact on hospital-acquired infections J Hosp Infect 2001 48 Suppl A S40 S46 11759025 10.1016/s0195-6701(01)90012-x

[B11] The Patient Inscurance LÖF http://www.patientforsakring.se/PRISS.html

[B12] Haynes AB Weiser TG Berry WR Lipsitz SR Breizat AH Dellinger EP Herbosa T Joseph S Kibatala PL Lapitan MC A surgical safety checklist to reduce morbidity and mortality in a global population N Engl J Med 2009 360 5 491 499 10.1056/NEJMsa0810119 19144931

[B13] Classen DC Evans RS Pestotnik SL Horn SD Menlove RL Burke JP The timing of prophylactic administration of antibiotics and the risk of surgical-wound infection N Eng J Med 1992 326 5 281 286 10.1056/NEJM199201303260501 1728731

[B14] Steinberg JP Braun BI Hellinger WC Kusek L Bozikis MR Bush AJ Dellinger EP Burke JP Simmons B Kritchevsky SB Timing of antimicrobial prophylaxis and the risk of surgical site infections: results from the Trial to Reduce Antimicrobial Prophylaxis Errors Ann Surg 2009 250 1 10 16 10.1097/SLA.0b013e3181ad5fca 19561486

[B15] Weber WP Marti WR Zwahlen M Misteli H Rosenthal R Reck S Fueglistaler P Bolli M Trampuz A Oertli D The timing of surgical antimicrobial prophylaxis Ann Surg 2008 247 6 918 926 10.1097/SLA.0b013e31816c3fec 18520217

[B16] Sax H Allegranzi B Chraiti MN Boyce J Larson E Pittet D The World Health Organization hand hygiene observation method Am J Infect Control 2009 37 10 827 834 10.1016/j.ajic.2009.07.003 20004812

[B17] The National Board of Health and Welfare´s regulations on basic hygiene in the Swedish health servicehttp://www.socialstyrelsen.se/publikationer2007/thenationalboard

[B18] Silverman D Interpreting Qualitative Data Method for Analysing Talk, Text and Interaction 2001 London: Sage

[B19] World medical association declaration of Helsinki Ethical principles for medical research involving human subjects J Postgrad Med 2002 48 3 206 208 12432198

[B20] Bachoura A Guitton TG Smith RM Vrahas MS Zurakowski D Ring D Infirmity and injury complexity are risk factors for surgical-site infection after operative fracture care Clin Orthop Relat Res 2011 469 9 2621 2630 10.1007/s11999-010-1737-2 21161736 PMC3148392

[B21] Culver DH Horan TC Gaynes RP Martone WJ Jarvis WR Emori TG Banerjee SN Edwards JR Tolson JS Henderson TS Surgical wound infection rates by wound class, operative procedure, and patient risk index. National Nosocomial Infections Surveillance System Am J Med 1991 91 3B 152S 157S 1656747 10.1016/0002-9343(91)90361-z

[B22] Charnley J Eftekhar N Postoperative infection in total prosthetic replacement arthroplasty of the hip-joint. With special reference to the bacterial content of the air of the operating room. Br J Surg 1969 56 9 641 649 10.1002/bjs.1800560902 5808372

[B23] Lidwell OM Lowbury EJL Whyte W Effect of ultraclean air in operating rooms on deep sepsis in the joint after total hip or knee replacement: A randomised study Br Med J 1982 285 6334 10 14 10.1136/bmj.285.6334.10 6805791 PMC1499116

[B24] Lidwell OM Lowbury EJL Whyte W Airborne contamination of wounds in joint replacement operations: the relationship to sepsis rates J Hosp Infect 1983 4 2 111 131 10.1016/0195-6701(83)90041-5 6195220

[B25] Kärrholm J Garellick G Rogmark C Herberts P Swedish Hip Arthroplasty Register Annual Report 2007 2007 In. Gothenburg: Department of Orthopeadics Sahlgrenska University Hospital

[B26] Suzuki TMS Smith WR Stahel PF Gillani SA Hak DJ Postoperative surgical site infection following acetabular fracture fixation Injury 2010 41 4 396 399 Epub 2009 10.1016/j.injury.2009.11.005 20004894

[B27] Acklin YP Widmer AF Renner RM Frei R Gross T Unexpectedly increased rate of surgical site infections following implant surgery for hip fractures: problem solution with the bundle approach Injury 2011 42 2 209 216 10.1016/j.injury.2010.09.039 21047637

[B28] Andersson AE Bergh I Karlsson J Eriksson BI Nilsson K Traffic flow in the operating room: An explorative and descriptive study on air quality during orthopedic trauma implant surgery Am J Infect Control 2012 10.1016/j.ajic.2011.09.015 22285652

[B29] Gruenberg MF Campaner GL Sola CA Ortolan EG Ultraclean air for prevention of postoperative infection after posterior spinal fusion with instrumentation: a comparison between surgeries performed with and without a vertical exponential filtered air-flow system Spine (Phila Pa 1976) 2004 29 20 2330 2334 10.1097/01.brs.0000142436.14735.53 15480149

[B30] Hansen D Krabs C Benner D Brauksiepe A Popp W Laminar air flow provides high air quality in the operating field even during real operating conditions, but personal protection seems to be necessary in operations with tissue combustion Int J Hyg Environ Health 2005 208 6 455 460 10.1016/j.ijheh.2005.08.008 16325554

[B31] Whyte W Hodgson R Tinkler J The importance of airborne bacterial contamination of wounds J Hosp Infect 1982 3 2 123 135 10.1016/0195-6701(82)90004-4 6181129

[B32] Tammelin A Domicel P Hambraeus A Ståhle E Dispersal of methicillin-resistant Staphylococcus epidermidis by staff in an operating suite for thoracic and cardiovascular surgery: Relation to skin carriage and clothing J Hosp Infect 2000 44 2 119 126 10.1053/jhin.1999.0665 10662562

[B33] Edmiston JCE Seabrook GR Cambria RA Brown KR Lewis BD Sommers JR Krepel CJ Wilson PJ Sinski S Towne JB Molecular epidemiology of microbial contamination in the operating room environment: Is there a risk for infection? Surgery 2005 138 4 573 582 10.1016/j.surg.2005.06.045 16269284

[B34] AlBuhairn B Hind D Hutchinson A Antibiotic prophylaxis for wound infections in total joint arthroplasty : A systematic review Journal of Bone and Joint Surgery - Series B 2008 90 7 915 919 10.1302/0301-620X.90B7.20498 18591602

[B35] Gillespie WJ Walenkamp GH Antibiotic prophylaxis for surgery for proximal femoral and other closed long bone fractures Cochrane database of systematic reviews (Online) 2010 3 3 3 CD000244 10.1002/14651858.CD000244.pub2 PMC7043359 20238310

[B36] Stefansdottir A Robertsson O W-Dahl A Kiernan S Gustafson P Lidgren L Inadequate timing of prophylactic antibiotics in orthopedic surgery We can do better. Acta Orthopaedica 2009 80 6 633 638 10.3109/17453670903316868 PMC2823303 19995312

[B37] Van Kasteren MEE ManniÃ«n J Ott A Kullberg BJ De Boer AS Gyssens IC Antibiotic prophylaxis and the risk of surgical site infections following total hip arthroplasty: Timely administration is the most important factor Clin Infect Dis 2007 44 7 921 927 10.1086/512192 17342642

[B38] Insler SR Sessler DI Perioperative Thermoregulation and Temperature Monitoring Anesthesiol Clin North America 2006 24 4 823 837 10.1016/j.atc.2006.09.001 17342966

[B39] Rajagopalan S Mascha E Na J Sessler DI The effects of mild perioperative hypothermia on blood loss and transfusion requirement Anesthesiology 2008 108 1 71 77 10.1097/01.anes.0000296719.73450.52 18156884

[B40] Frank SM Fleisher LA Breslow MJ Higgins MS Olson KF Kelly S Beattie C Perioperative maintenance of normothermia reduces the incidence of morbid cardiac events: A randomized clinical trial J Am Med Assoc 1997 277 14 1127 1134 10.1001/jama.1997.03540380041029 9087467

[B41] Leslie K Sessler DI Bjorksten AR Moayeri A Mild hypothermia alters propofol pharmacokinetics and increases the duration of action of atracurium Anesth Analg 1995 80 5 1007 1014 7726398 10.1097/00000539-199505000-00027

[B42] Moretti B Larocca AM Napoli C Martinelli D Paolillo L Cassano M Notarnicola A Moretti L Pesce V Active warming systems to maintain perioperative normothermia in hip replacement surgery: a therapeutic aid or a vector of infection? J Hosp Infect 2009 73 1 58 63 10.1016/j.jhin.2009.06.006 19646785

[B43] Memarzadeh F Manning AP Comparison of operating room ventilation systems in the protection of the surgical site ASHRAE Transactions 2002 2 3 15 108 PART

[B44] Burke JP Infection control - a problem for patient safety N Eng J Med 2003 348 7 651 656 10.1056/NEJMhpr020557 12584377

[B45] Givens CD WR: Catheter-associated urinary tract infections in surgical patients: a controlled study on the excess morbidity and costs J Urol 1980 124 5 646 648 7452793 10.1016/s0022-5347(17)55596-2

[B46] Krediet AC Kalkman CJ Bonten MJ Gigengack ACM Barach P Hand-hygiene practices in the operating theatre: An observational study Br J Anaesth 2011 107 4 553 558 10.1093/bja/aer162 21665900

[B47] Loftus RW Koff MD Burchman CC Schwartzman JD Thorum V Read ME Wood TA Beach ML Transmission of pathogenic bacterial organisms in the anesthesia work area Anesthesiology 2008 109 3 399 407 10.1097/ALN.0b013e318182c855 18719437

[B48] Loftus RW Muffly MK Brown JR Beach ML Koff MD Corwin HL Surgenor SD Kirkland KB Yeager MP Hand contamination of anesthesia providers is an important risk factor for intraoperative bacterial transmission Anesth Analg 2011 112 1 98 105 10.1213/ANE.0b013e3181e7ce18 20686007

[B49] Bodenheimer T The American health care system–the movement for improved quality in health care N Engl J Med 1999 340 6 488 492 10.1056/NEJM199902113400621 9971876

[B50] Stahel PF Sabel AL Victoroff MS Varnell J Lembitz A Boyle DJ Clarke TJ Smith WR Mehler PS Wrong-site and wrong-patient procedures in the universal protocol era: analysis of a prospective database of physician self-reported occurrences Arch Surg 2010 145 10 978 984 10.1001/archsurg.2010.185 20956767

[B51] Grol R Grimshaw J From best evidence to best practice: effective implementation of change in patients' care Lancet 2003 362 9391 1225 1230 10.1016/S0140-6736(03)14546-1 14568747

[B52] Polit DF Beck TC Nursing Research: Principles and Methods 2004 7 Philadelphia: Lippincott Williams& Wilkins

